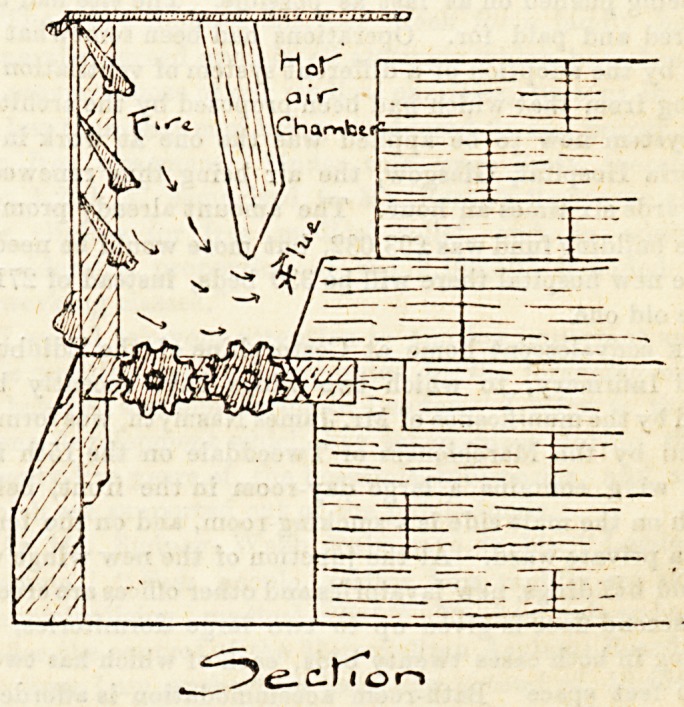# Smoke-Consuming Stoves

**Published:** 1893-04-08

**Authors:** 


### PRACTICAL DEPARTMENTS.

### SMOKE-CONSUMING STOVES.

While considering the comparative advantages of cooking by gas or by coal, we must not omit to mention, on behalf of the latter method, a new departure in that line which has lately been shown by the Economic Smokeless Fire Company at the recent exhibition at Islington, to which we referred last week. Messrs. Leggott and Marsh, of Bradford, are the patentees of "Smoke-consuming and Fuel-economising Kitchen Ranges," and their mode of construction is based on principles which, from a theoretical and scientific point of view, should ensure a practical success. We should explain, first, that the chief principle carried out in these ranges is that of a down-draught instead of an up-draught, whereby the smoke-consuming process is obtained, the smoke being forced down through the fuel, as indicated by the arrows in the accompanying diagram, and consumed during the operation. The plan is sufficiently simple, and the sectional plan, with a few words of explanation, will at once make the system clear to our readers. To begin with, the iron bars in front, "louvres," to give them the technical name, are made like the slats of a Venetian blind to open and shut, and are thus a means of regulating the rate of combustion. The bottom of the grate is fitted with two cogged rollers, which are manipulated by means of levers on the outside when it is necessary to remove any accumulation of ash. The fire is lighted from the top—an entire reversal of the usual plan—the coal placed at the bottom, then the wood, and lastly the paper. The powerful down-draught speedily causes the fire to burn well, and so successful does this method appear to be, that even in this early stage there is hardly any smoke to be seen, and as the fire burns up even this disappears. We have seen the range in working order less than an hour after its being lighted, when the amount of smoke was scarcely perceptible. The fire is kept well supplied from the top with small coal; and here comes in the economic advantages. Any kind of dust or rubbish may be used, the finest coal slack, or "smudge" as it is called in the North, being the fuel generally recommended, and as this can be obtained at 12s. 6d. per ton, the saving in the mere cost of the coal is very considerable. These ranges have been but a short time before the public, and, we believe, have not been put to practical use on a very large scale as yet, though in private houses they have, we understand, proved to be thoroughly successful. It is possible they may require further improvement in detail, but they are certainly the outcome of that increased scientific knowledge from which we hope so much in the future in its bearing on practical and domestic subjects, and if, where fire heat is preferred to gas, these stoves come into general use, it would surely mean an end to the great fog nuisance which is such a disgrace to our civiliastion. The system has hardly yet been satisfactorily applied to sitting-room fireplaces, though Messrs. Leggott and Marsh are now bringing it to bear in that direction. No doubt the mode of construction is not as applicable to sitting-room grates as to kitchen ranges, according to our received English ideas of the advantages of open fires, as they would necessarily have somewhat more of the character of a stove, but provided the heat obtained is equal to that of an open fire, a mere sentiment as to the superior blessing of the latter should not stand in the way of the adoption of a system which would be productive of immense good to the entire population.

## Figures and Tables

**Figure f1:**